# Mutational profiling of Chinese patients with thyroid cancer

**DOI:** 10.3389/fendo.2023.1156999

**Published:** 2023-07-03

**Authors:** Yaying Du, Shu Zhang, Gang Zhang, Jiaying Hu, Lianhua Zhao, Yuanyuan Xiong, Lu Shen, Rongrong Chen, Ke Ye, Yan Xu

**Affiliations:** ^1^ Department of Thyroid and Breast Surgery, Tongji Hospital, Tongji Medical College, Huazhong University of Science and Technology, Wuhan, China; ^2^ Department of Breast and Thyroid Surgery, Daping Hospital, Army Military Medical University, Chongqing, China; ^3^ Ultrasound Diagnostic Department, Daping Hospital, Army Military Medical University, Chongqing, China; ^4^ Department of Pathology, Daping Hospital, Army Military Medical University, Chongqing, China; ^5^ Geneplus-Beijing, Beijing, China; ^6^ Department of General Surgery, Xiangya Hospital, Central South University, Changsha, Hunan, China

**Keywords:** thyroid cancer, genetic landscape, *TERT* promoter mutations, the number of somatic mutations, clonal architecture

## Abstract

**Background:**

The incidence of thyroid cancer in China has rapidly increased in recent decades. As the genetic profiles of thyroid cancer vary dramatically between different geographical regions, a comprehensive genetic landscape of thyroid cancer in the Chinese population is urgently needed.

**Methods:**

We retrospectively included thyroid cancer patients from three Chinese medical centers between February 2015 and August 2020. To dissect the genomic profiling of these patients, we performed targeted next-generation sequencing on their tumor tissues using a 1,021-gene panel.

**Results:**

A total of 458 Chinese patients with thyroid cancer were enrolled, including four malignant histological subtypes arising from follicular epithelial thyroid cells. *BRAF* driver mutations were identified in 76.0% of patients, followed by *RET* rearrangements (7.6%) and *RAS* driver mutations (4.1%). Tumors with more somatic mutations correlated with worse clinical characteristics, including older age at diagnosis, less differentiation of tumor, larger tumor size, lymph node metastasis and distal metastasis. Subclonal *BRAF* mutations occurred in 20% (6/30) of patients and were frequent in poorly differentiated or anaplastic tumors (33.3% [2/6] vs. 4.2% [1/24], *P* = 0.09) and those with distal metastasis (50.0% [2/4] vs. 8.7% [2/23], *P* = 0.09). Tumors with *TERT* promoter mutations had significantly more somatic mutations (average: 6.5 vs. 1.8, *P* < 0.001). Moreover, *TERT* promoter mutations were not associated with lymph node metastasis but significantly associated with older age at diagnosis and poorly differentiated or anaplastic tumors, regardless of their clonal architecture.

**Conclusion:**

Our results shed light on the molecular pathogenesis and clinical characteristics of thyroid cancer in the Chinese population. The number of somatic mutations, *TERT* promoter mutations, and the clonal architecture of *BRAF* mutations should be considered in the risk stratification of thyroid cancer.

## 1 Introduction

Thyroid cancer is the most common type of endocrine cancer. In China, the average annual percent change in thyroid cancer incidence between 2000 and 2015 was 12.4% (1). With its increasing incidence, thyroid cancer is currently the seventh most common malignancy in the Chinese population and the fourth most common malignancy in Chinese women (2). Although the mortality rate of thyroid cancer is relatively low, the rate of disease recurrence is high, occurring in 25%–35% of patients (3). According to different cellular origins and characteristics, thyroid cancer is categorized into five histological types, including papillary thyroid cancer (PTC), follicular thyroid cancer (FTC), poorly differentiated thyroid cancer (PDTC), anaplastic thyroid cancer (ATC), and medullary thyroid cancer, with the first four types arising from follicular epithelial thyroid cells, and medullary thyroid cancer arising from parafollicular cells (4).

The molecular pathogenesis of most thyroid cancers involves the constitutive activation of mitogen-activated protein kinase (MAPK) and phosphatidylinositol-3 kinase/Akt (PI3K/AKT) signaling pathways, which leads to excessive cell growth, proliferation, and survival (4). The most common mutation is the T1799A point mutation of the *BRAF* gene, which results in V600E amino acid substitution and leads to constitutive activation of serine/threonine kinase and excessive activation of the MAPK signaling pathway (5). Point mutations of the *RAS* genes and rearrangements of the *RET* gene are two other common mutations in thyroid cancer, both upstream of *BRAF* and acting through the MAPK and PI3K/AKT pathways (5). These mutations appear to be almost mutually exclusive, and their prevalence significantly differs across different geographical regions (5–8). Dysregulation of the PI3K/AKT pathway, triggered by point mutations and/or copy number alterations of *PIK3CA*, *PTEN*, and *AKT1* genes, is reportedly crucial for the initiation of FTC and less differentiated tumors (5, 9, 10). *TERT* promoter mutations, which promote telomerase activity and lead to telomere length maintenance, have been recently reported and frequently appear in aggressive and undifferentiated cancers (11–14). Two common mutations of the *TERT* promoter have been documented: the C228T and C250T substitutions. *TERT* promoter mutations are accompanied by oncogenic driver mutations (15). They are subclonal in PTC and clonal in PDTC and ATC (15, 16).

Two large-scale studies have revealed the genetic landscape of thyroid cancer in the Chinese population, especially in PTC (15, 17). However, small gene panels targeting PTC were used in these two studies. The comprehensive genetic landscape of PTC using a large gene panel in a large-scale Chinese cohort is lacking. Furthermore, most studies only enrolled PTC patients (15, 17–19), the difference in mutational profiles between well-differentiated and poorly-differentiated thyroid cancer captured by a large gene panel remains exclusive. In this study, we dissected the genetic landscape of Chinese thyroid cancer by applying targeted next-generation sequencing (NGS) with a 1,021 gene panel in 458 patients with thyroid cancer. We comprehensively explored the genetic profiling of Chinese thyroid cancer and its association with clinical features. Given our large gene panel, genomic characteristics, such as the number of somatic mutations, and clonal architecture were evaluated. Our study expands the understanding of genetic characteristics in thyroid cancer, which might facilitate better management of this disease.

## 2 Materials and methods

### 2.1 Patients and clinicopathologic data

This is a retrospective study. A total of 458 Chinese patients (339 females and 119 males) with thyroid cancer were enrolled in this study. These patients received total or near-total thyroidectomy at Xiangya Hospital, Tongji Hospital and Daping Hospital between February 2015 and August 2020. This study was approved by the ethics committees of Daping Hospital, Army Military Medical University (No. 2018-39), Xiangya Hospital, Central South University (No. 2019030441) and Tongji Hospital, Tongji Medical College, Huazhong University of Science and Technology (No. TJ-C20180110), and all patients signed a written consent. The clinicopathologic data, including age at diagnosis, gender, personal history, specific surgical procedure, detailed pathologic diagnosis, tumor stage, pT stage, pN stage, pM stage and metastasis site, were collected from the patient’s medical record. The classification of thyroid cancer and disease stage were defined by the World Health Organization criteria and the eight edition of the American Joint Committee on Cancer staging system, respectively.

### 2.2 DNA extraction, targeted capture, and targeted next-generation sequencing

Tumor tissues of primary lesions or lymph node metastasis were collected at surgery. Frozen tumor tissues or formalin-fixed, paraffin-embedded specimens (FFPE) were collected for sequencing. Five milliliters of peripheral blood samples were collected after surgery. The genomic DNA from frozen tumor tissues was extracted using the Tissue gDNA exaction Kit (Qiagen, Hilden, Germany). DNA from FFPE was isolated using Maxwell^®^16 FFPE Plus LEV DNA Purification kit (Qiagen, Hilden, Germany). Peripheral blood leukocytes (PBLs) were separated to extract germline genomic DNA using QIAamp DNA Blood Mini Kit (Qiagen, Hilden, Germany). DNA concentration and quality were assessed using a Qubit fluorometer (Invitrogen, Carlsbad, CA USA) and the Qubit dsDNA HS (High Sensitivity) Assay Kit. Before library construction, 400–800 ng each of genomic DNA extracted from PBLs and tumor specimens was sheared into fragments at a 200–250 bp peak with a Covaris S2 ultrasonicator (Covaris, Woburn, MA, USA). The KAPA Library Preparation Kit (Kapa Biosystems, Wilmington, MA, USA) was used to prepare indexed Illumina NGS libraries. DNA libraries of the tumor and its paired germline were hybridized to a previously reported custom-designed panel, which covers ~ 1.5 Mbp of the genome and targets 1,021 cancer-related genes (**Supplementary Table 1**) (20). The hybridized libraries were sequenced using a 100-bp paired-end configuration on a DNBSEQ-T7RS sequencer (MGI Tech, Shenzhen, China). The minimal mean effective depth of coverage for tissue and germline DNA was 300×.

### 2.3 Tumor somatic variant calling

Terminal adaptor sequences and low-quality reads were removed with FASTP (21), remaining reads were mapped to the reference human genome (hg19) and aligned using the BURROWS–WHEELER ALIGNER (version 0.7.12-r1039) with default parameters. Duplicated reads were removed with the MARKDUPLICATES tool in PICARD (version 4.0.4.0; Broad Institute, Cambridge, MA, USA). REALDCALLER (v1.8.1; Geneplus-Beijing, inhouse) (20) and GATK (v3.6-0-g89b7209; Broad Institute) were employed to detect tumor somatic single nucleotide variants and small insertions and deletions. CONTRA (2.0.8) was used to call copy number variations (22). NCSV (v0.2.3; Geneplus-Beijing, in-house) was applied to identify structural variants (20). All candidate variants were manually confirmed with the integrative genomics viewer browser. Variants were filtered to exclude germline mutations in dbSNP and those that occur at a population frequency of > 1% in ExAc (v0.3.1) or 1000 Genomes Project. An in-house database of clonal hematopoiesis variants of > 10,000 pan-cancer patients and healthy individuals was used to filter clonal hematopoiesis-related variants (23).

### 2.4 Tumor mutation burden evaluation

The tumor mutation burden (TMB) was determined as the number of somatic nonsynonymous single nucleotide variants and small insertions/deletions per mega-base in the coding region (with VAF ≥ 0.03).

### 2.5 PyClone analysis and intratumoral heterogeneity evaluation

Samples with more than three somatic substitution/small insertions and deletions were applied to PyClone by default to analyze the clonal structure using a Bayesian clustering method (24). Cancer cell fraction was calculated with the mean of predicted cellular frequencies. The cluster with the highest mean VAF was identified as the clonal cluster, and mutations in this cluster were clonal mutations. Meanwhile, other clusters and mutations were considered subclonal. Intratumoral heterogeneity evaluation (ITH) was calculated by the number of subclonal mutations to all mutations.

### 2.6 Statistical analysis

R package of ClusterProfiler (25) was performed to assess the biological significance of the somatic mutations based on the enrichment analysis of Gene ontology (GO) and Kyoto Encyclopedia of Genes and Genomes (KEGG). Statistical analyses were performed using SPSS version 19.0 (SPSS Company, Chicago, IL). The Mann-Whiney U test and Student’s *t*-test were used for non-normally and normally distributed continuous variables, respectively. The Kruskal-Wallis test was used to compare non-normally distributed continuous variables among three or more independently sampled groups. A comparison of categorical variables was conducted with Pearson’s χ2 test or Fisher’s exact tests. Pearson’s correlation coefficient was used to compare the number of somatic mutations and TMB, and maximum somatic allele frequency. All statistical tests were performed with two-sided methods, and *P* < 0.05 was considered to indicate statistical significance.

## 3 Results

### 3.1 Landscape of somatic genetic alterations

A total of 458 thyroid cancer patients were enrolled (**Table 1**; **Supplementary Table 2**). The median age at diagnosis was 38 years (range, 4-84), with 74.0% (*n* = 339) females. Most patients had PTC (95.6%, *n* = 438), followed by ATC (2.2%, *n* = 10), FTC (1.3%, *n* = 6), and PDTC (0.9%, *n* = 4). Most had unilateral thyroid tumors (53.1%, *n* = 243) and pT stage of I (65.0%, *n* = 298). Cervical lymph node metastasis appeared in 237 patients (51.7%). Among 18 patients with distal metastasis, the lung was the most common metastasis site and appeared in 13 patients (72.2%), followed by bone (44.4%, *n* = 8) and chest wall (11.1%, *n* = 2).

**Table 1:** Clinical characteristics of 458 Chinese patients with thyroid cancer.

	*n* = 458	%
Age, years		
Mean (SD)	40.1 (12.6)	
Median (min-max)	38 (4-84)	
Gender		
Female	339	74.0
Male	119	26.0
Histology		
PTC	438	95.6
FTC	6	1.3
PDTC	4	0.9
ATC	10	2.2
Tumor site		
Unilateral	243	53.1
Bilateral	67	14.6
Unknown	148	32.3
Tumor stage		
I	356	77.7
II	27	5.9
III	2	0.5
IV	6	1.3
Unknown	67	14.6
pT stage		
1	298	65.0
2	27	5.9
3	15	3.3
4	10	2.2
Unknown	108	23.6
pN stage		
0	152	33.2
1	237	51.7
Unknown	69	15.1
pM stage		
0	380	83.0
1	18	3.9
Unknown	60	13.1
Metastastatic site		
Lung	13	2.8
Bone	8	1.7
Chest wall	2	0.4
Liver	1	0.2
Brain	1	0.2
Pleura	1	0.2
Kidney	1	0.2

**Figure 1:**
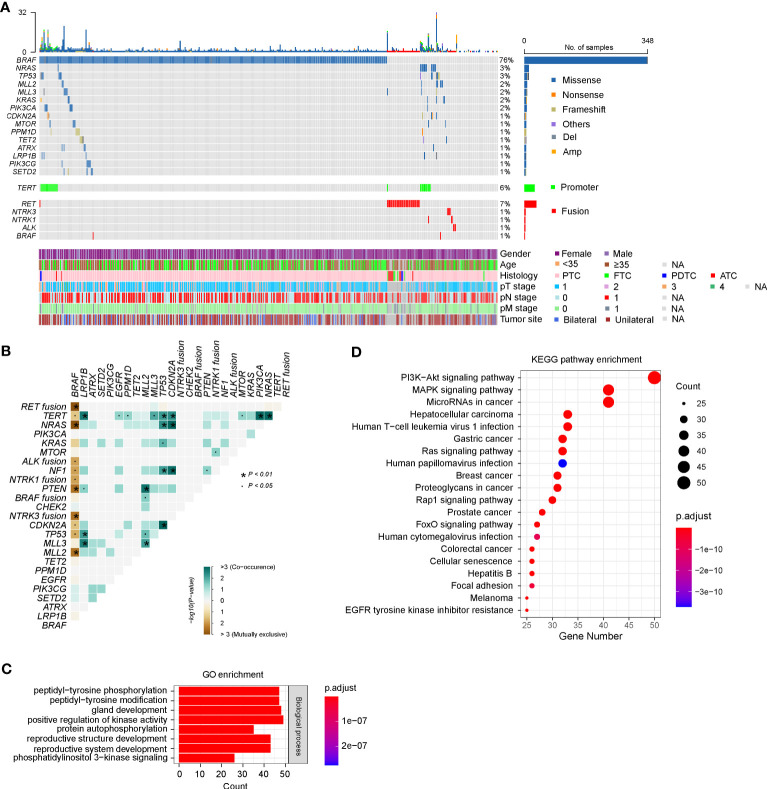
Genetic profile of 458 Chinese patients with thyroid cancer. **(A)** Heatmaps displaying the mutational landscape of 458 Chinese patients with thyroid cancer. Each column represents data from a single patient. The number of somatic mutations in each patient and the mutation frequency are shown at the top and right, respectively. The bottom heatmaps indicate key patient characteristics, including gender, age, histology, pT/N/M stage, and tumor site. **(B)** The coincident and exclusive associations across the top 25 mutated genes. The occurrences between *BRAF* and *NRAS*/*KRAS*/*NF1* mutations, and *RET*/*ALK*/*NTRK1*/*NTRK3* fusion were mostly mutually exclusive. **(C, D)** Gene Oncology (GO) **(C)** and Kyoto Encyclopedia of Genes and Genomes (KEGG) **(D)** analysis of all mutated genes in our cohort. PTC, papillary thyroid cancer; FTC, follicular thyroid cancer; PDTC, poorly differentiated thyroid cancer; ATC, anaplastic thyroid cancer. GO, Gene Ontology, KEGG, Kyoto Encyclopedia of Genes and Genomes.

According to the sequencing data, a total of 949 somatic alterations were identified in 436 patients, with an average of 2.1 alterations per patient. Among these alterations, 879 (92.6%) were substitution/small insertions and deletions, with 49 (5.2%) fusion/rearrangements, 16 (1.7%) gene amplifications and 5 (0.5%) gene deletions (**Figure 1A**; **Supplementary Table 2**). *BRAF* was identified as the most commonly mutated gene, which appeared in 348 (76.0%) patients (**Figure 1A**). Among them, 344 harbored the *BRAF V600E* substitution mutation, one had the *BRAF V600E* mutation/AGK-BRAF fusion co-occurrence, one harbored the *BRAF K601E*/*H608R* co-mutation, one had *BRAF V600E*/*G327W* co-mutation, and one had *PAK1*-*BRAF* fusion. *RAS* genes, including *KRAS* and *NRAS*, were also identified as frequently mutated and mutually exclusive with *BRAF* mutations, occurring in 19 (4.1%) patients (**Figure 1A**). Mutations in *PI3KCA*, *PTEN*, and *AKT1* genes, which are members of the PI3K/AKT pathway, were found in 7 (1.5%), 4 (0.9%) and 2 (0.4%) patients, respectively. Among them, *PTEN* mutations were mostly mutually exclusive with *BRAF* mutations (**Figure 1B**).

Eighteen types of gene fusion were identified in 48 PTC patients, and most were kinase-encoding genes (94.4%, *n* = 17). The most common fused gene was *RET*, which occurred in 35 cases (7.6%), followed by *NTRK3* (0.9%, *n* = 4), *NTRK1* (0.7%, *n* = 3), *ALK* (0.7%, *n* = 3) and *BRAF* (0.4%, *n* = 2) (**Supplementary Table 2**). Consistent with previous results, gene fusions were mutually exclusive with *BRAF* mutations (**Figure 1B**). Nevertheless, two PTCs concurrently harbored *BRAF V600E* substitution mutations and *AGK*-*BRAF*, and *NCOA4*-*RET* gene fusions, respectively. Among 17 kinase gene fusions, three novel gene fusions have not been documented, including two in-frame gene fusions between *RET* and other genes (*GRIPAP1* and intergenic region of *GRAMD3*) and one in-frame gene fusion between *BRAF* and *PAK1*. *GRIPAP1* encodes a guanine nucleotide exchange factor for the Ras family of small G proteins, which has been reported as a partner gene of *TFE3* in translocation renal cell carcinoma (26). In our study, the 5’ region of *GRIPAP1* (promoter–exon 26) was connected to the 3’ region of *RET* (intron 11–exon 20). *GRIPAP1-RET* was found in a female PTC patient, who only had this alteration. *RET* (exons 2–20) was fused to a point 75.0 kb upstream of the coding region of the *GRAMD3*, which encoded a GRAM domain and a transmembrane domain anchoring it to the endoplasmic reticulum (27). *GRAMD3* (intergenic)-*RET* gene fusion was identified in a female PTC patient, and she also harbored 16 other alterations, such as *CCDC6-RET* fusion. *PAK1* encodes a family member of serine/threonine p21-activating kinase and links RhoGTPases to cytoskeleton reorganization and nuclear signaling (28). The 5’ region of *PAK1* (promoter–intro 9) was fused to the 3’ region of *BRAF* (intron 8–exon 18). *PAK1*-*BRAF* was identified in a female PTC patient, who also had *ARID1A* and *MLL2* mutations concurrently.

The prevalence of *TERT* promoter (*TERTp*) mutations in our cohort was 6.3% (*n* = 29), including 5.2% (n = 24) *C228T* and 1.1% (n = 5) *C250T* substitutions (**Figure 1A**; **Supplementary Table 2**). The emergence of *TERTp* mutations is usually accompanied by oncogenic driver mutations, including *BRAF* (*n* = 17), *NRAS* (*n* = 7), *KRAS* (*n* = 1), *RET* (*n* = 1), and *NTRK1* (*n* = 1).

According to the GO enrichment, mutated genes of our cohort might be involved in the following common biological processes: peptidyl-tyrosine phosphorylation or modification, gland development, positive regulation of kinase activity, protein autophosphorylation, reproductive structure development, reproductive system development, and phosphatidylinositol 3-kinase signaling (**Figure 1C**). Further analyses using KEGG pathway annotations identified several pathways with significant representation in thyroid cancer, including PI3K/AKT, MAPK kinase, RAS, RAP1, and FoxO signaling pathways (**Figure 1D**).

### 3.2 Genomic features associated with lymph node metastasis, age at diagnosis and differentiation status in thyroid cancer

As thyroid cancer is associated with a high risk of lymph node metastasis (LNM) (29), we explored the genomic features associated with pathologic LNM in thyroid cancer. Compared with patients with non-LNM, patients with LNM were younger and had a higher proportion of males (**Supplementary Table 3**). To compare the mutational profiles between patients with and without LNM and avoid age and gender as potentially confounding variables, we matched these two groups artificially. We identified 198 out of the 237 patients from the LNM group that matched 152 patients with non-LNM. The matched LNM groups had a more advanced pT stage (**Supplementary Table 3**). No statistical differences were detected when comparing the frequency of altered genes between these two groups.

**Figure 2:**
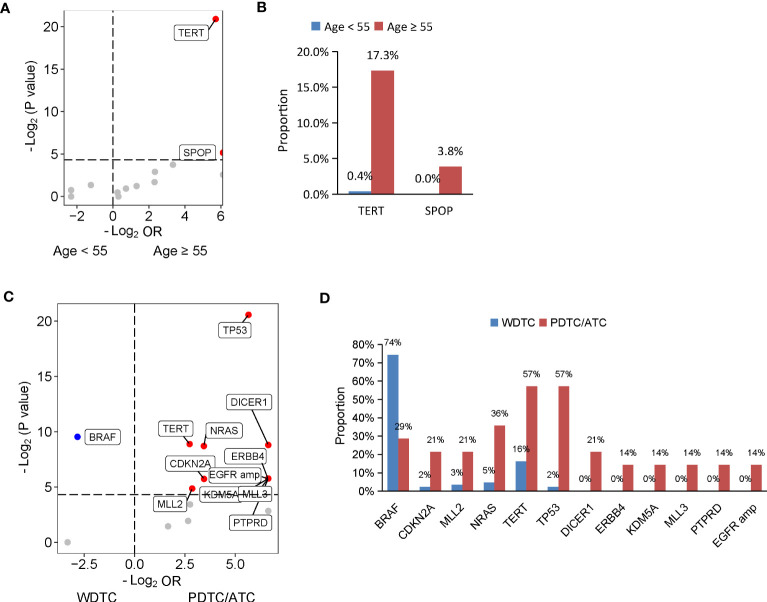
Genomic profiles associated with age at diagnosis and differentiation status in thyroid cancer. **(A, C)** Volcano plots showing the difference of genomic alterations between thyroid cancer patients with age at diagnosis < 55 and ≥ 55 years **(A)** and between patients with WDTC and PDTC/ATC **(B)**. WDTC, well-differentiated thyroid cancer; PDTC, poorly differentiated thyroid cancer; ATC, anaplastic thyroid cancer. **(B, D)** Histograms showing the prevalence of differentiated mutated genes shown in **(A)** and **(C)**, respectively.

Given the evidence that age at diagnosis is critical for risk stratification and 55 years is regarded as the cut-off to determine tumor stage (30), we then dissected the mutational features associated with age at diagnosis. A total of 365 patients diagnosed at age ≥ 55 years and 59 patients diagnosed at age < 55 years were included in our cohort. Compared with patients diagnosed at age < 55 years, patients diagnosed at age ≥ 55 years had a significantly lower proportion of PTC histology (**Supplementary Table 4**). Similarly, a matched comparison was performed to compare the difference in genomic alterations between young and old patients. As a result, 309 patients were matched, including 257 patients diagnosed at age < 55 years and 52 patients diagnosed at age ≥ 55 years, and two groups had comparable clinical characteristics (**Supplementary Table 4**). Two genes were altered at significantly higher frequencies in matched patients diagnosed at age ≥ 55 years than patients diagnosed at age < 55 years, *TERT* (17.3% vs. 0.4%; *P* < 0.001) and *SPOP* (3.8% vs. 0%; *P* = 0.03) (**Figures 2A, B**).

The degree of differentiation of tumors is related to their clinical behavior, and well-differentiated tumors tend to be less aggressive than poorly-differentiated ones. In our cohort, 444 patients had PTC or FTC, collectively known as well-differentiated thyroid cancers (WDTC), and 14 patients had PDTC or ATC, which are poorly differentiated or anaplastic tumors. Older age at diagnosis was found in patients with PDTC/ATC. To dig out the differentiation status-specific alterations, we performed a matched comparison between WDTC and PDTC/ATC groups. We identified 86 out of the 444 patients from the WDTC group that matched with 14 patients with PDTC/ATC (**Supplementary Table 5**). Patients with PDTC/ATC had more distal metastasis in the matched cohort, although statistical significance was not reached (*P* = 0.05) (**Supplementary Table 5**). A comparison of genomic profiles between the matched WDTC and PDTC/ATC tumors indicated that *BRAF* mutations were significantly enriched in WDTC tumors (74.4% vs. 28.6%; *P* = 0.001), and 11 genes were altered at significantly higher frequencies in PDTC/ATC tumors: *TP53* (57.1% vs. 2.3%; *P* < 0.001), *TERT* (57.1% vs. 16.3%, *P* = 0.002), *NRAS* (35.7% vs. 4.7%; *P* = 0.002), *DICER1* (21.4% vs. 0%; *P* = 0.002), *ERBB4* (14.3% vs. 0%; *P* = 0.02), *KDM5A* (14.3% vs. 0%; *P* = 0.02), *MLL3* (14.3% vs. 0%; *P* = 0.02), *PTPRD* (14.3% vs. 0%; *P* = 0.02), *CDKN2A* (21.4% vs. 2.3%; *P* = 0.02) and *MLL2* mutations (21.4% vs. 3.5%; *P* = 0.03), and *EGFR* amplification (14.3% vs. 0%; *P* = 0.02) (**Figures 2C, D**).

### 3.3 High somatic mutation numbers associated with poor clinical features

**Figure 3:**
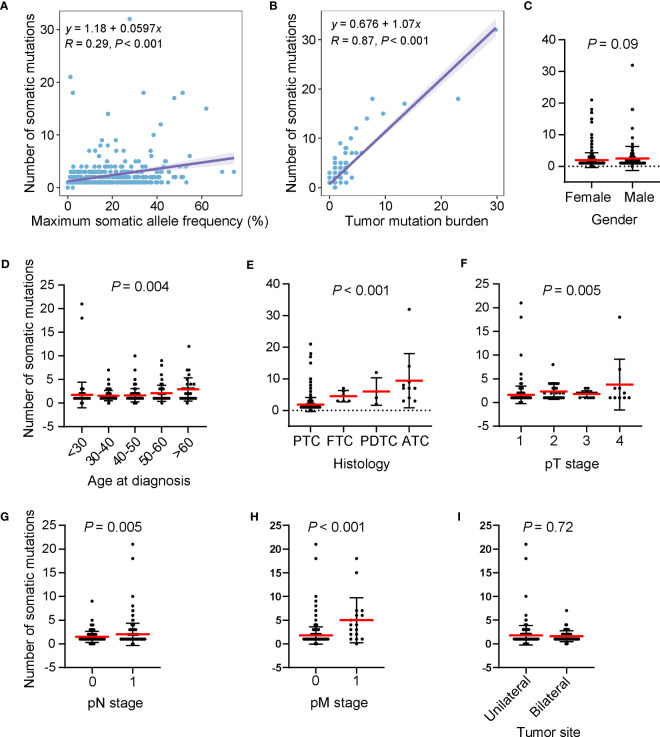
High somatic mutation numbers associated with poor clinical features. **(A, B)** Scatter plots showing the correlation between the number of somatic mutations and maximum somatic allele frequency **(A)** and tumor mutation burden **(B)**. **(C–I)** Differences in the number of somatic mutations by gender **(C)**, age at diagnosis **(D)**, histology **(E)** pT stage **(F)**, pN stage **(G)**, pM stage **(H)**, and tumor site **(I)**, respectively. Dots, whiskers and red lines indicate the number of somatic mutations, the Standard Error and the mean number of somatic mutations, respectively. PTC, papillary thyroid cancer; FTC, follicular thyroid cancer; PDTC, poorly differentiated thyroid cancer; ATC, anaplastic thyroid cancer.

The number of somatic mutations (including single nucleotide variants, small insertions/deletions, copy number change and structural variants) varied among patients, ranging from 0 to 32, with most patients harboring one mutation (53.5%, *n* = 245). The number of somatic mutations was positively correlated with the maximum somatic allele frequency (MSAF) and TMB (**Figures 3A, B**). We examined the relationship between the number of somatic mutations and clinical features and found that male patients had a mildly higher number of somatic mutations than in female patients (**Figure 3C**). The number of somatic mutations was positively correlated with age at diagnosis (**Figure 3D**). Patients with ATC had a significantly higher number of somatic mutations, followed by PDTC, FTC and PTC (**Figure 3E**). Furthermore, tumors with more advanced pT stage, LNM and distal metastasis had significantly higher number of somatic mutations when compared with their counterparts (**Figures 3F–H**). Tumor sites were not associated with the number of somatic mutations (**Figure 3I**). Together, higher somatic mutation numbers correlated with poor clinical features.

### 3.4 Clinical and genetic features of cancers carrying *TERT* promoter mutations

**Figure 4:**
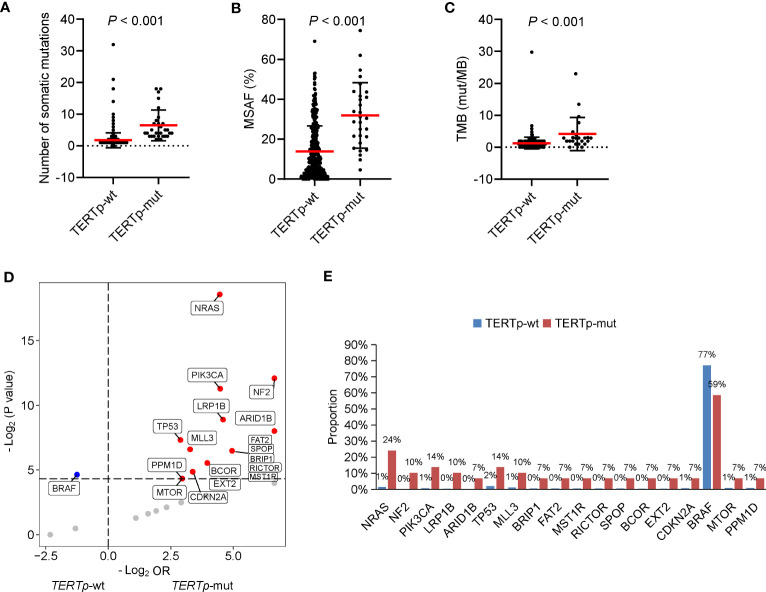
Clinical and genetic features of cancers carrying *TERTp* mutations. The differences in the number of somatic mutations **(A)**, maximum somatic allele frequency (MSAF) **(B)** and tumor mutation burden (TMB) **(C)** by *TERTp* mutation status. **(D)** Volcano plots showing the difference in genomic alterations between thyroid cancer patients with *TERTp*-wt and *TERTp*-mut. **(E)** Histograms showing the prevalence of differentiated mutated genes shown in **(D)**.

According to our above result, *TERTp* mutations were independently associated with older age at diagnosis and PDTC/ATC histology. Besides, patients with *TERTp* mutations had larger tumor sizes and more distal metastasis (**Table 2**), consistent with previous studies (11–14). Furthermore, the number of somatic mutations, MSAF and TMB were dramatically greater in *TERTp*-mut cancers than in *TERTp*-wt cancers (**Figures 4A–C**), supporting the idea that *TERTp*-mut cancers are aggressive. *BRAF* mutations were significantly enriched in *TERTp*-wt tumors (77.2% vs. 58.6%; *P* = 0.04). Seventeen somatic alterations were significantly enriched in *TERTp*-mut groups and mainly involved in PI3K/AKT or mTOR signaling pathways (**Figures 4D, E**).

**Table 2:** Comparison of clinical characteristics by *TERTp* status.

	*TERTp-*wt (*n* = 429)	*TERTp-*mut (*n* = 29)	*P* value
Age, years			<0.001
Mean (SD)	39.0 (11.5)	64.1 (11.6)	
Median (min-max)	37 (4-82)	65 (44-84)	
Gender, *n* (%)			0.03
Female	323 (75.3)	16 (55.2)	
Male	106 (24.7)	13 (44.8)	
Histology, *n* (%)			<0.001^a^
PTC	420 (97.9)	18 (62.1)	
FTC	3 (0.7)	3 (10.3)	
PDTC	2 (0.5)	2 (6.9)	
ATC	4 (0.9)	6 (20.7)	
Tumor site, *n* (%)			1.0
Unilateral	234 (54.5)	9 (31.0)	
Bilateral	65 (15.2)	2 (6.9)	
Unknown	130 (30.3)	18 (62.1)	
pT stage, *n* (%)			<0.001
1	296 (69.0)	2 (6.9)	
2	24 (5.6)	3 (10.3)	
3	15 (3.5)	0	
4	8 (1.9)	2 (6.9)	
Unknown	86 (20.0)	22 (75.9)	
pN stage, *n* (%)			0.09
0	150 (35.0)	2 (6.9)	
1	225 (52.4)	12 (41.4)	
Unknown	54 (12.6)	15 (51.7)	
pM stage, *n* (%)			<0.001
0	370 (86.2)	10 (34.5)	
1	8 (1.9)	10 (34.5)	
Unknown	51 (11.9)	9 (31.0)	

a Comparison between PTC/FTC and PDTC/ATC.

PTC, papillary thyroid cancer; FTC, follicular thyroid cancer; PDTC, poorly differentiated thyroid cancer; ATC, anaplastic thyroid cancer.

### 3.5 Correlations between mutation clonality and clinical characteristics

Conferring plasticity to evolving tumors, genetic ITH is related to cancer cell proliferation, invasion and resistance to therapy (31). Here, we analyzed the ITH by performing PyClone analysis on 45 available patients, as these cases harbored more than three mutations and were qualified for PyClone analysis. When grouping patients by the median of ITH, no differences in clinical and genomic profiles were observed between groups with high and low ITH (**Supplementary Table 6**).

Clonal architecture is fundamental for understanding tumor evolution and affects the efficacy of therapy and clinical outcome (32, 33). We explored the clonal architecture of *BRAF* mutation, the most frequently actionable mutation in thyroid cancer. A total of 30 patients with *BRAF V600E* (n = 29) or *K601E* (n = 1) mutations were included in the analysis of *BRAF* mutation clonal architecture (i.e., clonality). Clonal and subclonal *BRAF* mutations were found in 24 and 6 patients. Among patients with subclonal *BRAF* mutation, clonal *TP53* mutations were found in 2 PDTC patients. Patients with subclonal *BRAF* mutations had a higher proportion of PDTC/ATC and higher MSAF than those of patients with clonal *BRAF* mutations (**Table 3**), the absence of statistical significance may be due to the limited sample size. No significant co-mutations were found between these two groups.

**Table 3:** Comparison of clinical characteristics by *BRAF*/*TERTp* mutation clonality.

	Subclonal *BRAF* (n = 6)	Clonal *BRAF* (n = 24)	*P* value	Subclonal *TERT* (n = 13)	Clonal *TERT* (n = 6)	*P* value
**Age, years**			0.13			0.43
Mean (SD)	59.8 (7.9)	47.9 (16.5)		68.5 (5.2)	61.5 (14.4)	
Median (min-max)	59.5 (52-68)	44 (24-81)		66.5 (65-76)	55.5 (47-81)	
**Gender, *n***			0.63			0.35
Female	5 (83.3)	15 (62.5)		8 (61.5)	2 (33.3)	
Male	1 (16.7)	9 (37.5)		5 (38.5)	4 (66.7)	
**Histology, *n***			0.09^a^			0.33^a^
PTC	4 (66.7)	23 (95.8)		7 (53.8)	4 (66.6)	
FTC	0	0		0	1 (16.7)	
PDTC	2 (33.3)	0		2 (15.4)	0	
ATC	0	1 (4.2)		4 (30.8)	1 (16.7)	
**pT stage, *n* (%)**			0.25			0.66
1	0	14 (58.3)		0	1 (16.7)	
2	1 (16.7)	5 (20.8)		1 (7.7)	1 (16.7)	
4	0	2 (8.3)		1 (7.7)	1 (16.7)	
Unknown	5 (83.3)	3 (12.5)		11 (84.6)	3 (50.0)	
**pN stage, *n* (%)**			0.51			0.38
0	1 (16.7)	6 (25.0)		1 (7.7)	0	
1	1 (16.7)	16 (66.7)		2 (15.4)	5 (83.3)	
Unknown	4 (66.6)	2 (8.3)		10 (76.9)	1 (16.7)	
**pM stage, *n* (%)**			0.09			0.29
0	2 (33.3)	21 (87.5)		2 (15.4)	4 (66.7)	
1	2 (33.3)	2 (8.3)		5 (38.5)	2 (33.3)	
Unknown	2 (33.3)	1 (4.2)		6 (46.1)	0	
**No. of somatic mutations**			0.19			0.01
Mean (SD)	7.3 (2.9)	7.2 (5.5)		10.0 (5.3)	4.7 (1.2)	
Median (min-max)	7 (4-12)	4 (4-21)		8 (4-18)	4 (4-7)	
**TMB, mut/MB**			0.18			0.18
Mean (SD)	3.6 (1.1)	3.5 (4.8)		6.5 (6.3)	2.4 (0.9)	
Median (min-max)	3 (2.9-4.8)	2.4 (1.0-23.0)		3.4 (1.9-23.0)	2.9 (1.0-3.0)	
**MSAF, %**			0.08			0.52
Mean (SD)	35.5 (17.0)	21.3 (13.5)		33.4 (17.4)	27.6 (10.2)	
Median (min-max)	36 (15.3-54.6)	20.3 (1.2-51.4)		33.9 (9.6-62)	26.7 (14.1-43.6)	

a Comparison between PTC/FTC and PDTC/ATC.

PTC, papillary thyroid cancer; FTC, follicular thyroid cancer; PDTC, poorly differentiated thyroid cancer; ATC, anaplastic thyroid cancer.

Clonality analysis was performed on 19 patients with *TERTp* mutations. Interestingly, clonal *TERTp* mutation was found in 6 patients, including 4 patients with PTC, which differs from the previous result that *TERTp* mutation acts as a subclonal genetic alteration in patients with PTC (15). No difference in clinical features was found between these two groups (**Table 3**). The co-mutations were similar between patients with clonal and subclonal *TERTp* mutations. However, patients with clonal *TERTp* mutations had fewer somatic mutations than patients with subclonal *TERTp* mutations (**Table 3**).

## 4 Discussion

To uncover the molecular mechanisms underlying Chinese thyroid cancer, tissue samples from 458 Chinese patients with thyroid cancer were collected and subjected to targeted NGS to detect somatic mutations. Among all cases, 95.2% harbored at least one somatic mutation. Although our panel covered 1,021 genes, the mutation burden was pretty low (2.1 non-synonymous mutations per patient), supporting the quiet genome of thyroid cancer (7, 34). Notably, 4.8% of patients with thyroid cancer have no genetic alterations. Cancer is caused by genetic alterations and epigenetic alterations, such as DNA methylation and histone modifications. Therefore, large-scale studies on the epigenetic profile of thyroid cancer are necessary.

The cervical lymph node is the most common metastasis site of thyroid cancer, which is prone to present in 40-90% of thyroid cancer (35, 36). We performed genetic-clinical correlation analysis to explore cervical lymph node metastasis-specific genomic alterations. Our result indicated that no gene alterations were enriched in the patients with lymph-node metastasis. Since lateral cervical lymph-node metastasis has more aggressive clinical behavior than cervical central lymph-node metastasis. Cervical lateral lymph-node metastasis-specific genomic alterations are worth to be explored. Age at diagnosis is critical for risk stratification, and older age at diagnosis is related to more frequent disease recurrence and distant metastases (30). Our data showed that *TERTp* mutation and *SPOP* mutation were enriched in patients with older age at diagnosis, indicating that *TERTp* and *SPOP* mutation might contribute to the poor prognosis of older patients. Altered genes of *TERT*, *TP53* and *NRAS*, encoding effectors in the ErbB and PI3K/AKT pathways, were enriched in patients with PDTC/ATC than in patients with WDTC, suggesting that these gene may enhance the dedifferentiation process.

Our data showed that the number of somatic mutations was higher in patients with older age at diagnosis, poorly differentiated or anaplastic tumors, larger tumor size, those with lymph node metastasis, and those with distal metastasis, which is consistent with the aggressive behavior that higher mutation burden impart on cancers. Although *BRAF* mutation is a driver mutation in thyroid cancer (7), its clonality varies. Among patients included for the analysis of *BRAF* mutation clonality, subclonal *BRAF* mutation was identified in 20% of patients and associated with more aggressive behaviors of tumors. Of note, clonal *TP53* mutations were found in 33.3% (n = 2) patients with subclonal *BRAF* mutations, and these two patients had PDTC. Given the evidence that dedifferentiation of thyroid cancer was evolved from a subclone of WDTC and defects in DNA repair could play an important role in the dedifferentiation process (37, 38), subclonal *BRAF* mutation and clonal *TP53* mutations may be the manifestation of tumor evolution and associated with aggressiveness of tumors. Due to the limited sample size qualified for PyClone analysis, the clonality of other common genes, such as *RAS*, was not analyzed in our cohort. Further larger cohorts are warranted to explore it. Collectively, we proposed that the number of somatic mutations and *BRAF* mutation clonality should be added to the molecular risk stratification in thyroid cancer besides mutated genes.

By conducting combined analyses of genetic alterations and clinicopathological features, we found that *TERTp* mutational status defined thyroid cancer with poor clinicopathologic features, including older age, more advanced tumor stage, more distant metastasis, and aggressive cancer types. The occurrence of *TERTp* mutations is significantly accompanied by driver mutations in the PI3K/AKT pathway, in line with the higher prevalence of *TERTp* mutations in aggressive cancers, because constitutive activation of the PI3K/AKT pathway is distinguishable in less differentiated tumors (5, 9, 10). Moreover, cancers with *TERTp* alterations had higher somatic mutation numbers, MSAF and TMB, supporting its contribution to the intratumoral heterogeneity and evasiveness of cancers. According to previous studies, *TERTp* mutations are subclonal in PTC and clonal in PDTC/ATC (15, 16). In our cohort, four PTCs had clonal *TERTp* mutations and 6 PDTC/ATCs had subclonal *TERTp* mutations. Different methods for clonality analysis may lead to these divergences. The previous studies analyzed the clonality by directly comparing the frequency of *TERTp* mutations with their accompanied driver mutations or among different histology, and we applied PyClone to analyze the clonality. According to our result, patients with clonal *TERTp* mutations had similar clinical features to patients with subclonal *TERTp* mutations. These results indicated that *TERTp* mutations might render the evasiveness of tumors regardless of their clonality.

Although PTC is the most common thyroid cancer subtype, its genetic and clinical characteristics vary across different geographic regions (5–8). We compared the characteristics of our PTC cohort with two other datasets, including a previous Chinese cohort consisting of 355 patients with PTC (15) and the Cancer Genome Atlas (TCGA) dataset consisting of 496 American patients with PTC (7). Our data showed that the age at diagnosis of our cohort was younger than that of the other two cohorts. The percentage of lymph node metastasis in our cohort was mildly higher than in the previous Chinese cohort and significantly higher than in the TCGA cohort (**Supplementary Table 7**). The increase in younger age at diagnosis and lymph node metastases in our cohort might be due to the improved sensitivity of diagnostic tools (39). Moreover, increased exposure to environmental carcinogens, such as medical radiation, should be considered (40). A comparison of common driver gene mutations indicated that our cohort had a significantly higher prevalence of *BRAF* mutations than the other two cohorts and a lower prevalence of *RAS* mutations and kinase gene fusions than the TCGA cohort. Additionally, the prevalence of *TERTp* mutations in our cohort was comparable with the previous Chinses cohort and significantly lower than the TCGA cohort. Taken together, our results indicate the genetic heterogeneity of PTCs among ethnic lines and geographical regions exist. It is therefore necessary to provide the Chinese population with reasonable prevention and protection measures for thyroid cancer.

## 5 Conclusion

Our study identified three novel gene fusions and displayed significant correlations between genomic characteristics and clinical features in Chinese patients with thyroid cancer. *TERTp* mutations, a high number of somatic mutations and subclonal *BRAF* mutations may correlate with worse clinical features and should be considered in the risk stratification of thyroid cancer. Nevertheless, large prospective cohorts are warranted to validate it.

## Data availability statement

The variation data presented in the study are deposited in the Genome Sequence Archive (41) in National Genomics Data Center (42), China National Center for Bioinformation / Beijing Institute of Genomics, Chinese Academy of Sciences, under accession number GVM000545 that can be publicly accessible at http://bigd.big.ac.cn/gvm/getProjectDetail?project=GVM000545. Of note, the variation data of three patients were lacking, their alterations were collected from their sequencing reports.

## Ethics statement

The studies involving human participants were reviewed and approved by Xiangya Hospital, Tongji Hospital and Daping Hospital ethics committees. Written informed consent to participate in this study was provided by the participants’ legal guardian/next of kin.

## Author contributions

YX, KY, YD, SZ and RC conceived and designed the study. YD, SZ, GZ, JH and LZ collected the clinical information and organized the database. YYX, LS and RC were in charge of the data analysis and interpretation. YYX contributed to manuscript writing. All authors contributed to manuscript revision and approved the submitted version.
