# Supplementary material for: Mutational profiling of Chinese patients with thyroid cancer
Source: Front Endocrinol (Lausanne). 2023 Jul 3;14:1156999. doi: 10.3389/fendo.2023.1156999 (PMC10351985; doi:10.3389/fendo.2023.1156999)
Supplement: Supplementary file 3 [file Table_3.docx]

**Supplementary Table 3.** **Comparison of clinical characteristics by lymph node metastasis**

|  | **Non-LNM (*n* = 152)** | **LNM before matching (*n* = 237)** | ***P* value** | **LNM after matching (*n* = 198)** | ***P* value** |
| --- | --- | --- | --- | --- | --- |
| **Age, years** |  |  | 0.008 |  | 0.33 |
| Mean (SD) | 41.3 (11.7) | 38.0 (11.9) |  | 40.1 (11.0) |  |
| Median (min-max) | 41 (22-72) | 36 (4-81) |  | 38 (24-81) |  |
| **Gender, *n* (%)** |  |  | 0.06 |  | 0.44 |
| Female | 121 (79.6) | 168 (70.9) |  | 150 (75.8) |  |
| Male | 31 (20.4) | 69 (29.1) |  | 48 (24.2) |  |
| **Histology, *n* (%)** |  |  | 0.27 |  | 0.21 |
| PTC | 152 (100) | 231 (97.5) |  | 194 (98.0) |  |
| FTC | 0 | 2 (0.8) |  | 2 (1.0) |  |
| PDTC | 0 | 1 (0.4) |  | 0 |  |
| ATC | 0 | 3 (1.3) |  | 2 (1.0) |  |
| **Tumor site, *n* (%)** |  |  | 0.05 |  | 0.13 |
| Unilateral | 99 (65.1) | 119 (50.2) |  | 102 (51.5) |  |
| Bilateral | 20 (13.2) | 44 (18.6) |  | 34 (17.2) |  |
| Unknown | 33 (21.7) | 74 (31.2) |  | 62 (31.3) |  |
| **pT stage, *n* (%)** |  |  | 0.003 |  | 0.01 |
| 1 | 140 (92.1) | 158 (66.7) |  | 139 (70.2) |  |
| 2 | 8 (5.3) | 19 (8.0) |  | 17 (8.6) |  |
| 3 | 2 (1.3) | 13 (5.5) |  | 10 (5.1) |  |
| 4 | 1 (0.6) | 9 (3.8) |  | 6 (3.0) |  |
| Unknown | 1 (0.6) | 38 (16.0) |  | 26 (13.1) |  |

LNM, lymph node metastasis; PTC, papillary thyroid cancer; FTC, follicular thyroid cancer; PDTC, poorly differentiated thyroid cancer; ATC, anaplastic thyroid cancer.

**Supplementary Table 4. Comparison of clinical characteristics by age at diagnosis**

|  | **Age < 55 before matching (*n* = 365)** | **Age ≥ 55 before matching (*n* = 59)** | ***P* value** | **Age < 55 after matching (*n* = 257)** | **Age ≥ 55 after matching (*n* = 52)** | ***P* value** |
| --- | --- | --- | --- | --- | --- | --- |
| **Gender, *n* (%)** |  |  | 0.26 |  |  | 0.4 |
| Female | 274 (75.1) | 40 (67.8) |  | 198 (77.0) | 36 (69.2) |  |
| Male | 91 (24.9) | 19 (32.2) |  | 59 (23.0) | 16 (30.8) |  |
| **Histology, *n* (%)** |  |  | <0.001 |  |  | 1.0 |
| PTC | 359 (98.4) | 52 (88.1) |  | 257 (100) | 52 (100) |  |
| FTC | 4 (1.0) | 2 (3.4) |  | 0 | 0 |  |
| PDTC | 1 (0.3) | 2 (3.4) |  | 0 | 0 |  |
| ATC | 1 (0.3) | 3 (5.1) |  | 0 | 0 |  |
| **Tumor site, *n* (%)** |  |  | 1.0 |  |  | 1.0 |
| Unilateral | 206 (56.4) | 29 (49.2) |  | 152 (59.1) | 28 (53.8) |  |
| Bilateral | 58 (15.9) | 8 (13.6) |  | 42 (16.3) | 8 (15.4) |  |
| Unknown | 101 (27.7) | 22 (37.3) |  | 63 (24.5) | 16 (30.8) |  |
| **pT stage, *n* (%)** |  |  | 0.37 |  |  | 0.37 |
| 1 | 263 (72.0) | 34 (57.6) |  | 183 (71.2) | 34 (65.3) |  |
| 2 | 21 (5.8) | 6 (10.2) |  | 14 (5.4) | 6 (11.5) |  |
| 3 | 14 (3.8) | 1 (1.7) |  | 8 (3.1) | 1 (2.0) |  |
| 4 | 8 (2.2) | 1 (1.7) |  | 7 (2.7) | 1 (2.0) |  |
| Unknown | 59 (16.2) | 17 (28.8) |  | 45 (17.5) | 10 (19.2) |  |
| **pN stage, *n* (%)** |  |  | 0.07 |  |  | 1.0 |
| 0 | 129 (35.3) | 24 (40.7) |  | 128 (49.8) | 24 (46.2) |  |
| 1 | 205 (56.2) | 21 (35.6) |  | 101 (39.3) | 19 (36.5) |  |
| Unknown | 31 (8.5) | 14 (23.7) |  | 28 (10.9) | 9 (17.3) |  |
| **pM stage, *n* (%)** |  |  | 0.03 |  |  | 0.62 |
| 0 | 328 (89.9) | 44 (74.6) |  | 225 (87.5) | 42 (80.8) |  |
| 1 | 10 (2.7) | 5 (8.5) |  | 6 (2.3) | 2 (3.8) |  |
| Unknown | 27 (7.4) | 10 (16.9) |  | 26 (10.1) | 8 (15.4) |  |

PTC, papillary thyroid cancer; FTC, follicular thyroid cancer; PDTC, poorly differentiated thyroid cancer; ATC, anaplastic thyroid cancer.

**Supplementary Table 5. Comparison of clinical characteristics by differentiation status**

|  | **WDTC before matching (*n* = 444)** | **PDTC/ATC (*n* = 14)** | ***P* value** | **WDTC after matching (*n* = 86)** | ***P* value** |
| --- | --- | --- | --- | --- | --- |
| **Age, years** |  |  | <0.001 |  | 0.17 |
| Mean (SD) | 39.8 (12.2) | 61.7 (17.8) |  | 55.8 (10.5) |  |
| Median (min-max) | 38 (4-84) | 65 (41-82) |  | 55 (12-84) |  |
| **Gender, *n* (%)** |  |  | 0.37 |  | 0.53 |
| Female | 330 (74.3) | 9 (64.3) |  | 63 (73.3) |  |
| Male | 114 (25.7) | 5 (35.7) |  | 23 (26.7) |  |
| **pT stage, *n* (%)** |  |  | 0.98 |  | 0.97 |
| 1 | 297 (66.9) | 1 (7.1) |  | 59 (68.6) |  |
| 2 | 27 (6.1) | 0 |  | 8 (9.3) |  |
| 3 | 15 (3.4) | 0 |  | 1 (1.1) |  |
| 4 | 10 (2.2) | 0 |  | 4 (4.7) |  |
| Unknown | 95 (21.4) | 13 (92.9) |  | 14 (16.3) |  |
| **pN stage, *n* (%)** |  |  | 0.16 |  | 0.14 |
| 0 | 152 (34.2) | 0 |  | 34 (39.5) |  |
| 1 | 233 (52.5) | 4 (28.6) |  | 44 (51.2) |  |
| Unknown | 59 (13.3) | 10 (71.4) |  | 8 (9.3) |  |
| **pM stage, *n* (%)** |  |  | <0.001 |  | 0.05 |
| 0 | 378 (85.1) | 2 (14.3) |  | 71 (82.6) |  |
| 1 | 15 (3.4) | 3 (21.4) |  | 15 (17.4) |  |
| Unknown | 51 (11.5) | 9 (64.3) |  | 0 |  |
| **Tumor site, *n* (%)** |  |  | 0.12 |  | 0.17 |
| Unilateral | 242 (54.5) | 1 (7.1) |  | 40 (46.5) |  |
| Bilateral | 65 (14.6) | 2 (14.3) |  | 13 (15.1) |  |
| Unknown | 137 (30.9) | 11 (78.6) |  | 33 (38.4) |  |

WDTC, well-differentiated thyroid cancer; PDTC, poorly differentiated thyroid cancer; ATC, anaplastic thyroid cancer.

**Supplementary Table 6. Comparison of clinical characteristics by the degree of ITH**

|  | **ITH < 0.75 (*n* = 22)** | **ITH ≥ 0.75 (*n* = 23)** | ***P* value** |
| --- | --- | --- | --- |
| **Age, years** |  |  | 0.24 |
| Mean (SD) | 53.2 (16.3) | 46.3 (11.6) |  |
| Median (min-max) | 52 (29-81) | 46 (24-65) |  |
| **Gender, *n* (%)** |  |  | 0.12 |
| Female | 12 (54.5) | 18 (78.3) |  |
| Male | 10 (45.5) | 5 (21.7) |  |
| **Histology, *n* (%)** |  |  | 0.88 |
| PTC | 15 (68.2) | 16 (69.6) |  |
| FTC | 2 (9.1) | 1 (4.3) |  |
| PDTC | 1 (4.5) | 2 (8.7) |  |
| ATC | 4 (18.2) | 4 (17.4) |  |
| **Tumor site, *n* (%)** |  |  | 0.59 |
| Unilateral | 10 (45.5) | 9 (39.1) |  |
| Bilateral | 1 (4.5) | 3 (13.1) |  |
| Unknown | 11 (50.0) | 11 (47.8) |  |
| **pT stage, *n* (%)** |  |  | 0.79 |
| 1 | 5 (22.7) | 10 (43.5) |  |
| 2 | 4 (18.2) | 3 (13.1) |  |
| 4 | 1 (4.5) | 1 (4.3) |  |
| Unknown | 12 (54.6) | 9 (39.1) |  |
| **pN stage, *n* (%)** |  |  | 0.56 |
| 0 | 4 (18.2) | 4 (17.4) |  |
| 1 | 10 (45.4) | 11 (47.8) |  |
| Unknown | 8 (36.4) | 8 (34.8) |  |
| **pM stage, *n* (%)** |  |  | 0.72 |
| 0 | 13 (59.1) | 13 (56.5) |  |
| 1 | 6 (27.3) | 4 (17.4) |  |
| Unknown | 3 (13.6) | 6 (26.1) |  |

ITH, intratumoral heterogeneity; PTC, papillary thyroid cancer; FTC, follicular thyroid cancer; PDTC, poorly differentiated thyroid cancer; ATC, anaplastic thyroid cancer.

**Supplementary Table 7. Comparison of clinical and genetic features among different cohorts of PTC**

|  | **This study (n = 438)** | **A Chinese cohort (n = 355)** | ***P* value** | **TCGA (n = 496)** | ***P* value** |
| --- | --- | --- | --- | --- | --- |
| **Clinical features** |  |  |  |  |  |
| **Age, years** |  |  | <0.001 |  | <0.001 |
| Mean | 39.6 | 44.2 |  | 46.8 |  |
| Median | 38 | 44 |  | 46 |  |
| **Gender, *n* (%)** |  |  | 0.08 |  | 0.76 |
| Female | 325 (74.2) | 281 (79.6) |  | 342 (73.2) |  |
| Male | 113 (25.8) | 72 (20.4) |  | 125 (26.8) |  |
| **pN stage, *n* (%)** |  |  | 0.07 |  | 0.001 |
| 0 | 152 (36.7) | 138 (46.6) |  | 214 (50.8) |  |
| 1 | 231 (60.3) | 158 (53.4) |  | 207 (49.2) |  |
| **Genetic variants** |  |  |  |  |  |
| ***BRAF*, *n* (%)** |  |  | 0.046 |  | <0.001 |
| Mutant | 344 (78.5) | 257 (72.4) |  | 240 (59.7) |  |
| Wild type | 94 (21.5) | 98 (27.6) |  | 162 (40.3) |  |
| ***RAS*, *n* (%)** |  |  | 0.49 |  | <0.001 |
| Mutant | 9 (2.1) | 10 (2.8) |  | 52 (12.9) |  |
| Wild type | 429 (97.9) | 345 (97.2) |  | 350 (87.1) |  |
| ***TERT*, *n* (%)** |  |  | 0.10 |  | 0.003 |
| Mutant | 18 (4.1) | 7 (2.0) |  | 36 (9.4) |  |
| Wild type | 420 (95.9) | 348 (98.0) |  | 348 (90.6) |  |
| **Kinase gene fusion, *n* (%)** |  |  | 0.19 |  | 0.05 |
| Mutant | 47 (10.7) | 49 (13.8) |  | 74 (15.3) |  |
| Wild type | 391 (89.3) | 306 (86.2) |  | 414 (84.7) |  |

TCGA: The Cancer Genome Atlas. TCGA cohort was reported in (1); and a Chinese cohort was reported in (2).

1. Cancer Genome Atlas Research N. Integrated Genomic Characterization of Papillary Thyroid Carcinoma. *Cell* (2014) 159(3):676-90. Epub 2014/11/25. doi: 10.1016/j.cell.2014.09.050.

2. Liang J, Cai W, Feng D, Teng H, Mao F, Jiang Y, et al. Genetic Landscape of Papillary Thyroid Carcinoma in the Chinese Population. *J Pathol* (2018) 244(2):215-26. Epub 2017/11/17. doi: 10.1002/path.5005.
